# Green Extraction of Antioxidant-Rich Flavonoids from *Fagonia cretica* Using Deep Eutectic Solvents

**DOI:** 10.3390/molecules30040813

**Published:** 2025-02-10

**Authors:** Jafar Khan, Sajjad Asaf, Lubna, Ashraf M. M. Abdelbacki, Rahmatullah Jan, Kyung-Min Kim

**Affiliations:** 1 Key Laboratory of Forest Plant Ecology, Northeast Forestry University, Harbin 150040, China; 2 Natural and Medical Science Research Center, University of Nizwa, Nizwa 616, Oman; sajadasif2000@gmail.com (S.A.); lubnabilal68@gmail.com (L.); 3 Deanship of Skills Development, King Saud University, P.O. Box 2455, Riyadh 11451, Saudi Arabia; aabdelbacki@ksu.edu.sa; 4 Coastal Agriculture Research Institute, Kyungpook National University, Daegu 41566, Republic of Korea; 5 Division of Plant Biosciences, School of Applied Biosciences, College of Agriculture and Life Science, Kyungpook National University, Daegu 41566, Republic of Korea

**Keywords:** antioxidant activity, deep eutectic solvents, flavonoid extraction, *Fagonia cretica*, sustainable recovery

## Abstract

This study optimized the extraction of flavonoids from *Fagonia cretica* using deep eutectic solvents (DESs), focusing on key factors such as the type of DES used, molar ratio, water content, solid/liquid ratio, extraction temperature, and time. Among six DESs tested, the betaine–acetic acid combination exhibited the highest extraction efficiency, attributed to its low viscosity (4.98 mPa·s). Optimal extraction conditions were determined to be a 1:4 molar ratio of betaine to acetic acid, a 25% water content, a solid/liquid ratio of 1:60 g/mL, an extraction temperature of 50 °C, and an extraction time of 30 min. Under these conditions, the flavonoid yield was maximized while preserving bioactivity. Antioxidant assays revealed that flavonoids extracted with DESs exhibited superior scavenging activity against DPPH and hydroxyl radical compared to ethanol-extracted flavonoids, highlighting DESs’ potential to enhance antioxidant properties. The recyclability of DESs was demonstrated using ultracapacitor porous activated carbon, achieving an 89.78% recovery efficiency. The reused DES maintained a high flavonoid extraction yield, retaining 92% efficiency after six cycles, emphasizing its sustainability and cost-effectiveness. This study establishes DES-based extraction as an environmentally friendly and efficient approach for isolating flavonoids with strong antioxidant properties, offering significant advantages in green chemistry and bioactive compound recovery.

## 1. Introduction

*Fagonia cretica*, a woody herb from the family Zygophyllaceae, thrives exclusively in the warm climates of Africa and Asia. Known for its bitter and sour flavors, it has gained a reputation for its therapeutic potential against a wide range of ailments, including inflammatory, neurological, hepatic, and hematological conditions [1]. This species, along with other members of the Fagonia genus, exhibits a diverse set of medicinal properties such as anti-asthmatic, antiseptic, antidotal, anti-dysenteric, anti-hepatotoxic, fever-reducing, diuretic, and pain-relieving effects [2]. Phytochemical investigations have identified a variety of chemical constituents in Fagonia species, including flavonoids, fatty acids, alkaloids, proteins, and amino acids [3]. These components have demonstrated a broad spectrum of bioactivities, such as antimicrobial, anticancer, antioxidant, and antihemorrhagic effects [4]. Among these, flavonoids stand out as crucial plant pigments, functioning as natural compounds and secondary metabolites [5]. Flavonoids are a key class of natural compounds with various phenolic structures, and they are extracted from natural sources [6]. These natural compounds protect plants from UV light, protect fat from oxidation, and protect enzymes and vitamins in plants [7]. They are widely acknowledged for their health-enhancing properties, including antioxidant, anti-inflammatory, antibacterial, anticancer, antiviral, and hypotensive effects, among others [8]. DESs have been used to extract various flavonoid compounds. Consequently, the efficient extraction of flavonoids from *F. cretica* is of significant interest. Traditional solid–liquid extraction techniques are commonly employed for obtaining flavonoids from natural sources [9]. The choice of solvent is a critical factor in these processes as it significantly influences the yield and quality of the extracted flavonoids [10].

Traditional organic solvents, including methanol, ethanol, acetone, and ethyl acetate, are commonly employed to isolate bioactive compounds from plants. However, their use raises sustainability concerns due to their toxicity, flammability, and limited biodegradability. Additionally, recycling these solvents from extraction residues is difficult, which can result in negative environmental impacts [11]. Hence, there is a strong push to develop sustainable and eco-friendly solvents for natural product extraction. DESs have gained attention as an effective and sustainable substitute for traditional organic solvent. They are cost-effective, easy to prepare, and convenient to store, and they exhibit properties like low toxicity, biodegradability, and a long shelf life. DESs are environmentally friendly and sustainable solvents characterized by their non-toxic and non-volatile nature. They exhibit unique properties, including high viscosity and a low vapor pressure, which make them safe and effective for various applications [12]. Additionally, DESs are known for their excellent recyclability and stability, further enhancing their appeal as a green alternative to traditional solvents in numerous industrial and research processes [12]. Due to physiochemical properties, such as density, viscosity, freezing temperature, polarity, surface tension, and conductivity, DESs are used as a potential solvent in industries. The physiochemical properties of DEGs can be tailored by varying the concentrations of hydrogen bond acceptors (HBAs) and hydrogen bond donors (HBDs) and adjusting their molar ratios [13]. In addition to the choice of HBA and HBD, factors such as their molar ratio, purity, temperature, water content, and the preparation method significantly influence the properties of DESs [14]. The pressure–volume–temperature (PVT) data for the densities of DESs are essential for designing equipment and processes, analyzing liquid–liquid equilibria, and optimizing mass transfer. These data also play a vital role in developing equations of state, creating predictive models, and calculating thermodynamic properties such as viscosity, thermal expansion coefficients, and isothermal compressibility [15]. The viscosity of DESs is a critical property due to its industrial relevance, influencing their suitability as reaction media [13]. Temperature-dependent viscosity data are essential for equipment design, understanding activation energy, and analyzing mass transport processes [16]. These characteristics have drawn considerable attention across various fields, including catalysis, organic synthesis, electrochemistry, and materials science, as well as for environmentally friendly extraction processes [17]. DESs are created by heating and mixing a hydrogen bond acceptor (HBA) with a hydrogen bond donor (HBD) until a clear solution is achieved. Choline chloride is a widely used HBA in the preparation of DESs, while HBDs often include alcohols, acids, sugar, and amines [18].

Despite the extensive use of choline chloride in extracting bioactive compounds from flowers, fruits, and roots [19], its high viscosity and tendency to crystallize can limit its extraction efficiency [20]. Therefore, expanding the range of available HBAs is crucial for enhancing the flexibility and efficiency of DES-based extractions. Acids, when employed as HBDs, often offer a more environmentally friendly profile and produce lower-viscosity mixtures comparison with alcohols, sugars, and amines [21]. Thus, synthesizing DESs using unique HBAs and acid-based HBDs represents a promising approach to improving extraction outcomes. The effectiveness of the extraction depends significantly on the solvent used [20]. In this study, flavonoids are the primary target compounds, and different solvents (DESs, methanol, ethanol, and water) are tested to extract them selectively. It is anticipated that various extraction solvents will yield different flavonoid profiles, which in turn will exhibit unique antioxidant activities. Compounds such as quercetin, kaempferol, and catechin, which can be obtained using DESs, have demonstrated similar antioxidant properties, such as the ability to scavenge DPPH and hydroxyl radicals when compared to ethanol extracts [22]. The recovery of solvents is essential for optimizing the extraction process more viable and in line with green chemistry principles [23]. However, conventional recovery methods for DESs are often inadequate. Carbon-based adsorption materials, known for their exact surface area, small particle size, excellent stability, and strong adsorption capabilities, offer significant potential for the efficient recovery of DESs. These materials are simple to handle and may improve the sustainability of extraction by allowing for solvent reuse [24]. The objective of this research was to determine a safe and effective DES-based technique for extracting flavonoids from *Fagonia cretica*. The optimization of extraction conditions was the first step, followed by an assessment of the antioxidant properties of the extracts. Finally, we explored the feasibility of solvent recovery and reuse. Six types of DESs were prepared using glucose, choline chloride, betaine, L-proline, sucrose, and glycerol as HBAs, paired with acetic acid as the HBD. Here, we focused on modifying HBAs rather than HBDs because the selection of HBA plays a more significant role in influencing the solvent’s polarity, viscosity, and overall extraction efficiency. The choice of HBA directly impacts the strength and nature of hydrogen bonding interactions, which are crucial for solubilizing different compounds. On the other hand, acetic acid was selected as the HBD due to its well-established compatibility with various HBAs and its favorable properties for extraction processes. We decided to keep acetic acid as the HBD to isolate and assess the impact of different HBAs on extraction efficiency. Future studies could explore variations in both the HBA and HBD to further refine and optimize the DES formulations. The key extraction parameters optimized included the DES molar ratios, water content, solid–liquid ratio, extraction temperature, and time. After optimization, the antioxidant activity of the flavonoid-rich extracts was evaluated. Four types of carbon adsorption materials were tested for their ability to recover DES, and the potential for reusing the recovered solvent was examined.

## 2. Materials and Methods

### 2.1. Plant Material and Chemicals

Rutin (98% purity) was sourced from Macklin Biochemical Co., Ltd. (Shanghai, China). Additional chemicals, including choline chloride (99%), betaine (96%), L-proline (99%), glucose (98%), acetic acid (98%), sodium hydroxide (99%), sodium nitrite (99%), aluminum nitrate (99%), ultra-high-capacity porous activated carbon (UCPAC), graphene (GPH), multi-walled carbon nanotubes (MWCNTs), mesoporous carbon (MPC), DPPH, ethanol, ferrous sulfate, and salicylic acid (99%), were obtained from Aladdin Reagent Co., Ltd. (Shanghai, China). The plant material, *Fagonia cretica*, was harvested from Pakistan. The biomass was dried at 35 °C using an electric oven and then ground into a fine powder and filtered using a 40-mesh sieve. The resulting powder was stored at 4 °C to preserve its quality for future experimental procedures.

### 2.2. Preparation of DESs

Deep eutectic solvents (DESs) were synthesized by a standard procedure involving heating and stirring. A round-bottom conical flask was employed to create binary mixtures of HBAs such as glucose, choline chloride, betaine, L-proline, sucrose, and glycerol with acetic acid serving as the HBD at specific molar ratios. To each mixture, 3% water was added. The mixture was subsequently stirred continuously using magnetic stirrer in a water bath set at 90 °C until a uniform, clear liquid was achieved. A detailed overview of the prepared DESs can be found in Table 1.

### 2.3. Flavonoid Extraction by Using DESs

A measured amount of 0.10 g of finely ground *F. cretica* powder was placed into 15 mL tubes containing 10 mL of a deep eutectic solvent (DES) solution, which was diluted with 30% water. The extraction process was conducted using an ultrasonic device set to 50 °C for 30 min. Following the extraction process, the mixture was subjected to centrifugation at 6000 rpm for 15 min. The upper layer was carefully separated and stored in Eppendorf tubes for subsequent analysis.

### 2.4. Total Flavonoid Contents

Rutin was utilized as a reference to quantify the flavonoid concentration extracted from *Fagonia cretica*. The experimental method was based on an established protocol with slight modifications [25]. A standard solution of 50 mL was prepared by mixing 5 mg rutin into 70% ethanol. Various volumes of this standard solution were then added into the tube, and an additional 0.3 mL of 10% NaNO_2_ solution was added. After mixing, we added 10% of aluminum nitrate (0.3 mL) and mixed it by inverting the tube a few times and then keeping it for 6 min. Subsequently, we added 4% NaOH (4 mL) to the mixture and adjusted the total volume to 10 mL by adding 70% ethanol. The solution was mixed by shaking for 15 min. The absorbance was recorded at 510 nm using a UV-Vis spectrophotometer (Agilent Cary 60, Santa Clara, CA, USA). The calibration curve was constructed using the standard solution data. The absorbance of the extracted sample was measured using the same protocol, and the calibration curve was used to evaluate the flavonoid concentration in the extracts. The extraction yield was calculated using the following formula below:

Extraction yield (mg/g) = (n × C × V)/M,

where V is the volume of the extracted solution, n is the dilution factor, C is the concentration of the solution extracted, and M is the mass of the dried sample.

### 2.5. Optimizing Extraction Flavonoids

The extraction efficiency of flavonoids served as a key indicator to refine the extraction conditions. A series of experiments were conducted, varying several parameters. These included testing six different types of deep eutectic solvents (DESs): Glu-AA, ChCl-AA, Be-AA, L-pro-AA, Suc-AA, and Gly-AA. Additionally, six molar ratios of the DESs were examined (1:1, 1:2, 1:3, 1:4, 1:5, and 1:6). The influence of the water content was explored across five levels (15%, 25%, 35%, 55%, and 75%). Experiments also evaluated seven different solid-to-liquid ratios, specifically 1:10, 1:20, 1:30, 1:40, 1:50, 1:60, and 1:70 g/mL. Various extraction temperatures were tested (20, 30, 40, 50, and 60 °C), as well as extraction durations ranging from 10 to 60 min (10, 20, 30, 40, and 60 min). The outcomes of these experiments were utilized to determine the optimal extraction parameters.

### 2.6. Evaluation of DPPH Free Radical Scavenging Ability

To assess the antioxidant potential of the sample, its capacity to neutralize free radicals was assessed. The extraction yield of flavonoids served as a crucial factor in optimizing the extraction method. A modified version of the DPPH radical scavenging assay, adapted from [26], was used to measure the antioxidant properties of the flavonoid extracts. In this procedure, 2 mL of a 0.2 mmol/L DPPH solution was mixed with 2 mL of extracts obtained using DES3 (different ratio) and ethanol. The mixtures were incubated for 30 min in the absence of light to allow the reaction to proceed. Absorbance at 517 nm was measured, and the DPPH scavenging activity was determined using the following equation:

DPPH radical activity = 1 − (A1 − A2/A0) × 100

where A1 represents the absorbance of blink, A2 represents the absorbance of the investigated sample, and A0 is the absorbance of the control sample without any extract.

### 2.7. Recovery and Reuse of Deep Eutectic Solvents

In this study, various carbon-based adsorbent materials were utilized to recover and recycle deep eutectic solvents (DESs) for continued use in subsequent experiments. The material used in the recovery process included ultra-high-capacity porous activated carbon, graphene, multi-walled carbon nanotubes, and mesoporous carbon. The process began by mixing 5 mL of the DES extract with 100 mg of the carbon adsorbent, followed by shaking for 10 min. The mixture was then subjected to centrifugation at 6000× *g* for 15 min and passed through a 0.22 µm filter paper. The purified DES was collected, and the absorbance was evaluated using a spectrophotometer (Agilent Cary 60 UV-Vis). Flavonoid separation and DES purification were achieved through the adsorption of flavonoids onto the carbon materials. The purified DES was subsequently reused for extracting flavonoids from fresh *F. cretica* powder following the same extraction protocol. This recovery and reuse process was performed six times, and the results were examined to assess the efficiency of the recovered DES for flavonoid extraction.

### 2.8. Hydroxyl Radical Searching Assessment

The hydroxyl radical scavenging activity was evaluated following the protocol described in [27]. Different amount of the DES and ethanol extract were prepared. Each tube contained 2 mL of the sample solution, containing ferrous sulfate, hydrogen peroxide, and salicylic acid, 1 mL each. The mixtures were thoroughly shaken and incubated in a water bath at 37 °C for one hour. Following incubation, absorbance was measured at 510 nm, and the scavenging activity was calculated using the following formula:

$$\mathrm{Hydroxyl}\;\mathrm{radical}\;\mathrm{scavenging}\;\mathrm{activity}=1-\left(\frac{A1-A2}{A0}\right)\times 100\%$$

where A1 is the absorbance of the sample containing an active compound, A2 is the absorbance of the blank reagent, and A0 is the absorbance of the blank control sample.

### 2.9. Statistical Analysis

The data were analyzed statistically using SPSS software, version 18.0. The mean values and standard deviations were calculated based on three replicates. A one-way analysis of variance (ANOVA) was applied to identify statistically significant differences, considering a *p*-value of less than 0.05 as indicative of significant variation. Graphical representations were generated using GraphPad prism 10.4.1.

## 3. Results

### 3.1. Screening of Suitable DESs for the Isolation of Target Compounds

**Figure 1:**
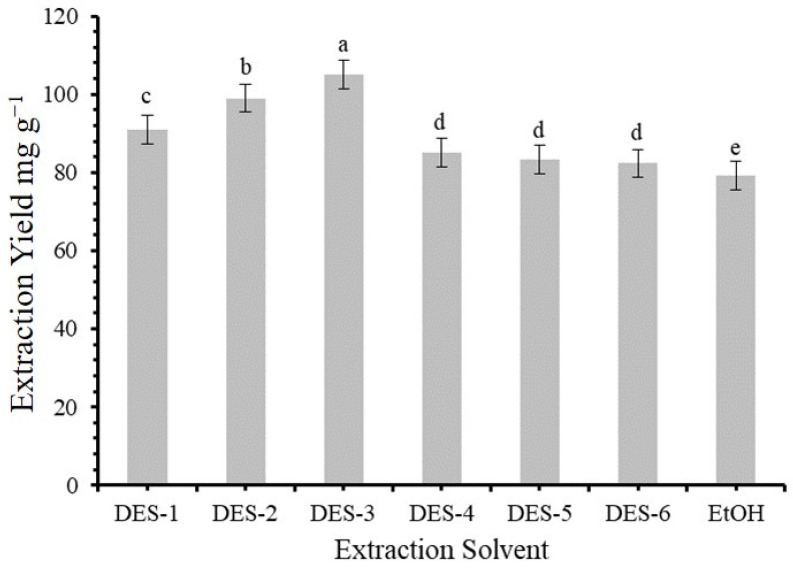
Comparison of extraction yields (mg·g^−1^) obtained using various extraction solvents, including six deep eutectic solvents (DES-1 to DES-6 represent glucose, choline chloride, betaine, L-proline, sucrose, and glycerol, respectively) and ethanol (EtOH). Bars represent mean values ± standard error. Statistically significant differences (*p* < 0.05) among solvents, as determined by post hoc analysis, are indicated by different letters above bars.

One of the critical factors in solid–liquid extraction is choosing the best effective deep eutectic solvent (DES) [26]. In this study, we used glucose, choline chloride, betaine, L-proline, sucrose, and glycerol as HBAs and acetic acid as the HBD. Consequently, six different DESs were prepared: Glu-AA, ChCl-AA, Be-AA, L-pro-AA, Suc-AA, and Gly-AA. The results are presented in Figure 1. Among these, the Bet-AA combination showed the highest flavonoid extraction efficiency from the plant biomass. This is likely because Bet-AA exhibits the lowest viscosity among the solvents tested, which enhances the extraction process. The viscosity of the six DESs Glu-AA, ChCl-AA, Be-AA, L-pro-AA, Suc-AA, and Gly-AA was calculated using a rotating viscometer, yielding values of 5.30, 6.10, 4.98, 6.09, 5.99, and 7.04 mPa·s, respectively. During extraction, the diffusion of the target compounds from the solid phase into the liquid phase depends on the viscosity of the solvent. A lower viscosity leads to faster diffusion, improving the extraction efficiency. Therefore, the higher viscosity of some DESs correlated with a reduced flavonoid extraction capacity. As a result, Bet-AA was chosen as the preferred solvent for subsequent extraction experiments. 

### 3.2. An Evaluation of the Suitable Molarity

**Figure 2:**
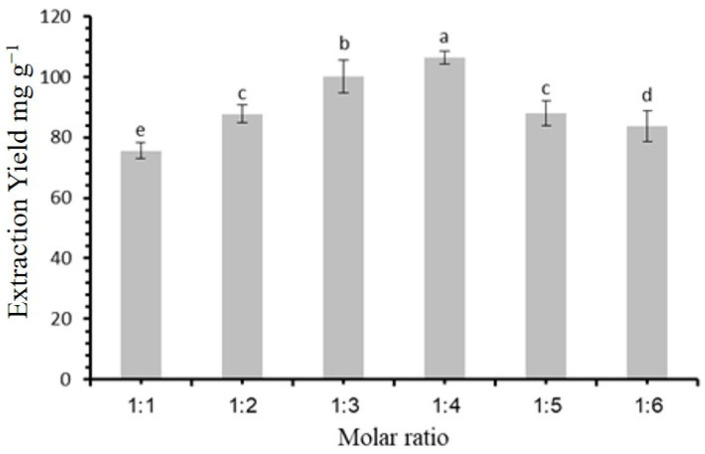
Extraction yield (mg·g⁻^1^) as function of molar ratio of deep eutectic solvent components. Bars represent mean values ± standard error. Statistically significant differences (*p* < 0.05) among molar ratio, as determined by post hoc analysis, are indicated by different letters above bars.

The molar ratio between the HBA and HBD has a key role in defining the chemical and physical characteristics of DESs, which in turn affects the extraction efficiency [28]. To investigate this, we used various molar ratios of betaine to acetic acid (1:1, 1:2, 1:3, 1:4, 1:5, and 1:6). The outcome, presented in Figure 2, show that the maximum extraction yield was attained with a 1:4 molar ratio of betaine to acetic acid. Based on these findings, a 1:4 ratio was chosen for the extraction of flavonoids from *F. cretica* in further experiments.

### 3.3. An Evaluation of the Optimum Water Concentration

**Figure 3:**
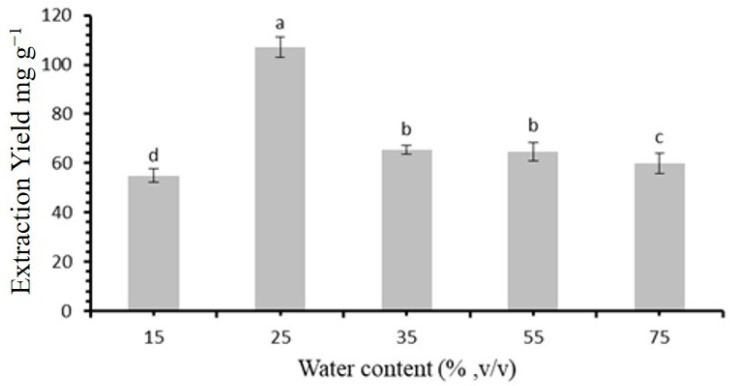
Effect of water content (% *v*/*v*) in deep eutectic solvent on extraction yield (mg·g⁻^1^). Bars represent mean values ± standard error. Statistically significant differences (*p* < 0.05) among water content levels, as determined by post hoc analysis, are indicated by different letters above bars.

The water content is another crucial factor in the extraction process. In this study, various water concentrations (15%, 25%, 35%, 55%, and 75%) were tested to determine the optimal water level in the extraction medium. The results depicted in Figure 3 demonstrate that the yield increased initially, peaking at a 25% water content, before declining as the content continued to rise; the initial increase can be attributed to the role of water in lowering the viscosity of the DES, thereby improving the flavonoid extraction efficiency, as previously discussed. However, beyond this optimal level, excessive water disrupts the hydrogen bonding between the HBA and HBD, ultimately reducing the solvents’ effectiveness in extracting flavonoids [29]. Therefore, a 25% water content was selected for further experiments.

### 3.4. Screening of the Best Solid/Liquid Ratio

**Figure 4:**
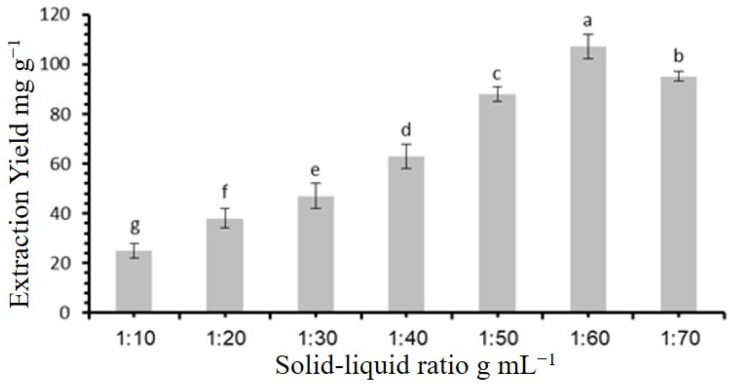
Effect of solid/liquid ratio on flavonoid extraction yield. Statistically significant differences (*p* < 0.05) among solid/liquid ratio, as determined by post hoc analysis, are indicated by different letters above bars.

The impact of different solid/liquid ratios (1:10, 1:20, 1:30, 1:40, 1:50, 1:60, and 1:70 g/mL) on the flavonoid extraction efficiency was evaluated. The findings, shown in Figure 4, reveal that the extraction yield increased slowly, reaching its peak at a solid/liquid ratio of 1:60 g/mL. Beyond this ratio, the yield slightly decreased. Consequently, a solid/liquid ratio of 1:60 g/mL was chosen for further flavonoid extraction experiments from *Fagonia cretica*.

### 3.5. Effect of Temperature on Extraction Yield

**Figure 5:**
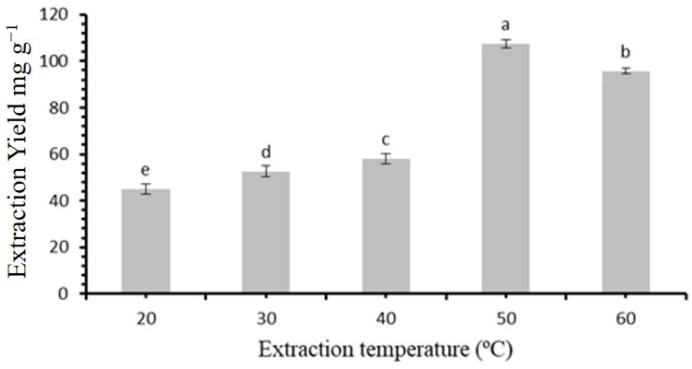
Temperature regulate the extraction yield of flavonoides. Statistically significant differences (*p* < 0.05) among extraction temperatures, as determined by the post hoc analysis, are indicated by different letters above the bars.

As part of the optimization process, the influence of various extraction temperatures (20, 30, 40, 50, and 60 °C) was assessed, and the results are presented in Figure 5. The extraction yield increased progressively with the temperature, peaking at 50 °C. However, when the temperature exceeded 50 °C, the yield began to decline. An increase in temperature enhances molecular diffusion, which accelerates the mass transfer of target compounds, thereby improving the flavonoid extraction efficiency. Nonetheless, excessively high temperatures can compromise the integrity of flavonoid structures, potentially reducing their bioactivity and lowering the extraction yields [26]. Hence, a temperature of 50 °C was established as the suitable condition for the further extraction trials.

### 3.6. Effect of Time on Extraction Yield

**Figure 6:**
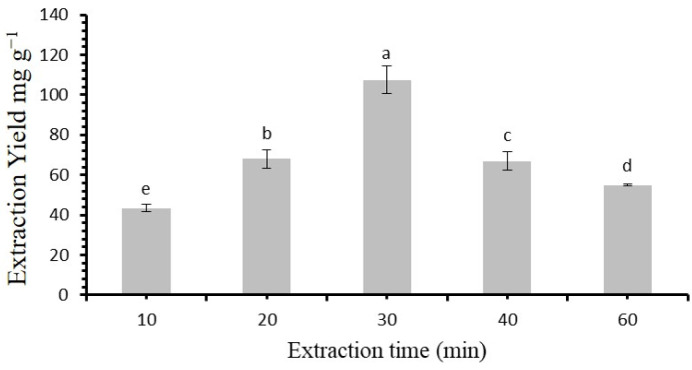
Time of extraction regulate flavonoids yield. Statistically significant differences (*p* < 0.05) among extraction times, as determined by post hoc analysis, are indicated by different letters above bars.

The time variation effects on extraction yield of flavonoids was further examined, with times ranging from 10 to 60 min (Figure 6). The yield increased steadily from 10 to 30 min. However, after 30 min, the yield began to decline. This initial rise in extraction efficiency is expected, as more flavonoids are able to diffuse from the solid material as the extraction time progresses. On the other hand, prolonged extraction times can lead to the degradation and structural damage of flavonoids, which can reduce the overall yield [10]. In light of these findings, an extraction time of 30 minutes was selected as the suitable time for further extraction trails.

### 3.7. Evaluation of Antioxidant Activity of Deep Eutectic Solvents and Ethanol Extracts

**Figure 7:**
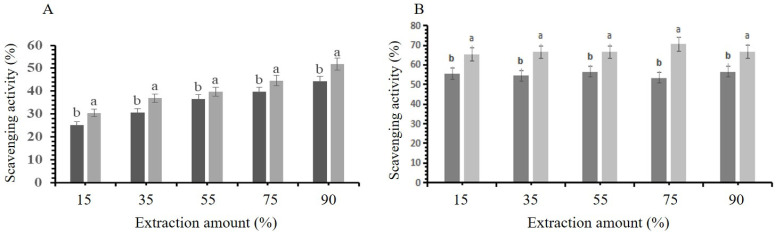
DPPH and hydroxyl free radical scavenging activity for different extraction amounts. (**A**) Graph illustrating the free radical scavenging capacity of extract obtained using DESs and ethanoles. (**B**) Graph illustrating the hydroxyl radical scavenging activity f extract produced with DESs and ethanoles. Statistically significant differences (*p* < 0.05) among ethanol and different DES amounts, as determined by post hoc analysis, are indicated by different letters above bars. Dark gray bars represent ethanol data and light gray shows DES data.

Oxidation is a primary factor contributing to food spoilage, leading to the loss of vital nutrients such as vitamins, enzymatic browning, the formation of undesirable flavors, and potentially harmful oxidation by-products. To evaluate the antioxidant potential of the DES and ethanol extracts, the scavenging activity against DPPH and hydroxyl radicals was assessed (Figure 7A,B). The results clearly demonstrated that the flavonoids possessed a significant capacity to neutralize these radicals. Notably, under identical extraction conditions, the flavonoids extracted using DESs demonstrated superior scavenging activity compared to those obtained with ethanol. These findings suggest that DESs and ethanol may isolate various types and amount of flavonoids from *F. cretica*, with DES-based extracts showing stronger antioxidant properties.

### 3.8. Recovery and Reuse of Deep Eutectic Solvents

**Figure 8:**
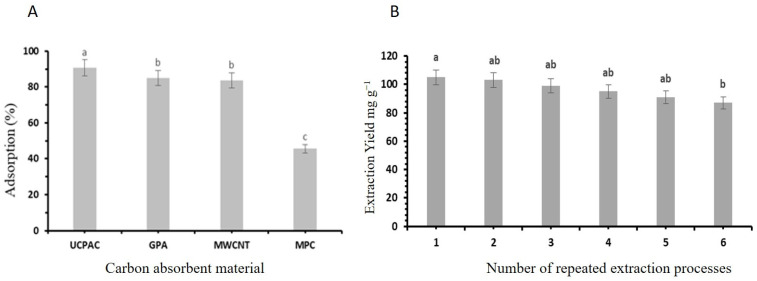
(**A**) Graphical illustration shows absorption percentage obtained by using four different corban absorption materials. (**B**) Graphical illustration shows extraction yield by reusing DESs. Statistically significant differences (*p* < 0.05) in the carbon absorbance material (**A**) and the number of repeated extraction processes (**B**), as determined by the post hoc analysis, are indicated by different letters above the bars.

From an economic standpoint, the recycling potential of deep eutectic solvents (DESs) is a crucial factor. The potential for reusing DESs in the extraction process for targeted compounds from *F. cretica* was evaluated under the same conditions used for the initial extraction. After centrifugation, the mixture was partitioned into two phases. The aqueous phase, which contained the phenolic compounds, was collected for further analysis, while the DES phase, which had a lower phenolic content, was retained for reuse in subsequent extraction cycles. UCPAC, GPA, MWCNT, and MPC were employed to recover the used DES (Figure 8A), with ultracapacitor porous activated carbon (UCPAC) demonstrating the highest absorption efficiency (89.78%). This can be attributed to UCPAC’s large surface area, small particle size, and high adsorption capacity [30]. The regained DESs was used again to extract flavonoids from fresh *F. cretica* powder. The flavonoid isolation level was assessed as described earlier, and the solvent was recycled for another extraction cycle. The results of these cyclic extractions are shown in Figure 8B. Despite slight losses in the DES volume during each cycle, the extraction yields remained significantly high, even after six cycles. By the sixth cycle, the yield remained 92% of the value. These findings suggest that the DES can be effectively recovered and reused without significant performance loss, demonstrating its superior recycling potential compared to other methods. In conclusion, the selected DES offers an environmentally friendly, recyclable, and reusable extraction system.

## 4. Discussion

Flavonoids are bioactive phytochemicals with diverse pharmacological properties, including antioxidant, anti-inflammatory, antibacterial, antiviral, and anti-tumor activities, making them highly valuable for pharmaceutical, food, and cosmetic applications [12]. The extraction of flavonoids from *F. cretica* was systematically optimized through a series of experiments focusing on key extraction parameters. These parameters included the selection of suitable DESs, the determination of the ideal molar ratio, the assessment of the appropriate water content, the evaluation of the ideal solid–liquid ratio, and the consideration of the influence of temperature and extraction time. Specific DESs, including glucose, choline chloride, betaine, L-proline, sucrose, and glycerol, were selected as HBAs combined with acetic acid as the HBD. In the screening process, each of the DESs mentioned above comprises different combinations of HBAs and HBDs. Among these combinations, betaine–acetic acid (Bet-AA) emerged as the most effective for flavonoid extraction, exhibiting a statistically significant increase in yield compared to the other DESs. The optimized DES, Bet-AA, demonstrated a flavonoid extraction yield of 105.4 mg/g, showcasing its superior efficiency [31]. A recent study reported that the NaDES mixture of betaine and urea is highly effective for extracting phenolic compounds with strong antioxidant properties and yielded the highest phenolic content and antioxidant activity [32]. Another study showed that the extraction efficiency, assessed through the total phenolic compound and flavonoid contents, showed that choline chloride–acetic acid and choline chloride–lactic acid were the most effective, with all tested DESs outperforming ethanol and exhibiting high antioxidant activity [33]. Furthermore, a betaine-based DES combined with glycol demonstrated superior efficiency in extracting chlorogenic acids from spent coffee grounds (waste by-product of coffee) compared to the conventional organic solvent [34]. This choice was substantiated by the low viscosity of Bet-AA, which facilitated efficient flavonoid extraction. Viscosity proved to be a critical factor, as higher viscosity hindered the diffusion rate of target compounds, leading to a reduced extraction efficiency. In recent years, the use of DESs for extracting polyphenolic compounds from various food matrices has gained significant attention [35]. These solvents have proven to be versatile in applications such as extraction processes, food analyses, enzymatic reactions, purification, recycling, and fermentation. Specifically, DESs have demonstrated their ability to isolate both hydrophilic and lipophilic compounds of interest [36]. For example, DESs have been successfully employed to recover anthocyanins from jaboticaba (*Myrciaria cauliflora*) fruit waste, tannic acid from onion peels, and phlorotannins from algae. Moreover, they have been utilized to extract phenylethanes and phenylpropanoids from *Rhodiola rosea* L., steroidal saponins from *Dioscoreae nipponicae* rhizoma, and polyphenols from olive pomace, among other bioresources [37,38,39,40]. These examples highlight the broad applicability of DESs in efficiently recovering valuable bioactive compounds from diverse natural sources, making them a promising tool for sustainable and efficient extraction methods.

The measured viscosities of all DESs provide valuable context to this finding [41]. The molar ratio of betaine to acetic acid was further assessed, ranging from 1:1 to 1:6. The maximum extraction yield was observed at a molar ratio of 1:4, indicating an optimal balance between betaine and acetic acid for efficient flavonoid extraction. At this ratio, the extraction yield reached its highest value of 105.4 mg/g (*p* < 0.05) [41]. Another crucial parameter considered was the water content in the extraction media. The experiments revealed a progressive increase in the extraction yield with a water content of up to 25%, after which further increments led to a gradual decrease. This initial rise in extraction efficiency is attributed to the reduction in DES viscosity, which aids in flavonoid extraction. However, excessive water content can disrupt the hydrogen bonding between HBA and HBD, diminishing the extraction capability (*p* < 0.05). Similar trends were also recorded in the case of *Garcinia mangostana* L, as reported in [42]. The study also evaluated various solid/liquid ratios, leading to the selection of a ratio of 1:60 g mL. This choice optimizes extraction efficiency while avoiding diminishing returns. It resulted in a statistically significant increase in extraction yield compared to other ratios (*p* < 0.05). Temperature was revealed as a critical factor for flavonoid extraction. The experiments indicated an increase in extraction yield with rising temperatures, up to an optimal value of 50 °C, which is similar to those recorded in [43]. Beyond this point, a decrease in the extraction yield was observed. This trend is attributed to enhanced molecular diffusion and mass transfer of target compounds at higher temperatures. In contrast, excessively high temperatures can result into to structural damage and the breakdown of flavonoids [44].

The antioxidant capacity, a critical factor in food preservation, was a focal point of this study. Flavonoids extracted with DESs exhibited higher scavenging activity compared to ethanol extracts. In a recent study, it was reported that the DES significantly enhanced the extraction of total flavonoids and antioxidants from *Rudbeckia hirta* flowers compared with conventional extraction techniques [12]. This indicates the potential superiority of DES-based extraction methods in extracting potentially bioactive compounds. The statistically significant increase in scavenging activity further supports this observation (*p* < 0.05) [22]. The study addressed the economic viability of DESs through their recycling capability. Notably, UCPAC demonstrated the highest absorption capacity, resulting in a statistically significant increase in DES recovery compared to other carbon adsorption materials (*p* < 0.05). The consistently high extraction yields, even after multiple cycles, suggest the sustainability and cost-effectiveness of this approach [5].

## 5. Conclusions

This study developed an eco-friendly and efficient ultrasound-assisted extraction method for flavonoids from *F. cretica* using deep eutectic solvents (DESs). The optimal yield (105.4 mg/g) was achieved with a DES composed of betaine and acetic acid, surpassing conventional ethanol extraction in antioxidant potential. Notably, the DES was successfully recovered and reused, demonstrating sustainability and efficiency. The extracted flavonoids exhibited enhanced oxidative stability, underscoring the method’s value. This innovative approach highlights DESs as a sustainable alternative for extracting bioactive compounds, promoting environmental and practical advantages in solid–liquid extractions.

## Figures and Tables

**Table 1:** DESs used for flavonoid extraction.

Solvents	HBAs	HBD	M/Ratio	Appearance
DES1	Glucose	Acetic acid	1:1	Transparent
DES2	Choline chloride	Acetic acid	1:1	Transparent
DES3	Betaine	Acetic acid	1:1, 1:2, 1:3, 1:4, 1:5, 1:6	Transparent
DES4	L-proline	Acetic acid	1:1	Transparent
DES5	Sucrose	Acetic acid	1:1	Transparent
DES6	Glycerol	Acetic acid	1:1	Transparent

Note: HBA, hydrogen bond acceptor; HBD, hydrogen bond donor; M, molar mixture; and DES, deep eutectic solvent.

## Data Availability

Data is contained within the article.
